# Exploring the phenotype and genotype of multi-drug resistant *Klebsiella pneumoniae* harbouring *bla*_CTX-M_ group extended-spectrum *β*-lactamases recovered from paediatric clinical cases in Shenzhen, China

**DOI:** 10.1186/s12941-019-0331-z

**Published:** 2019-11-05

**Authors:** Sandip Patil, Xiaowen Chen, Feiqiu Wen

**Affiliations:** 1 Department of Haematology and Oncology, Shenzhen Children’s Hospital, 7019 Yitian Road, District Futian, Shenzhen, 518038 Guangdong Province China; 2 Shenzhen Institutes of Advanced Technology, Chinese Academy of Sciences, Shenzhen, 518055 Guangdong Province China; 3 Paediatric Research Institute, Shenzhen Children’s Hospital, 7019 Yitian Road, District Futian, Shenzhen, 518038 Guangdong Province China

**Keywords:** *Klebsiella pneumoniae*, ESBLs, Antimicrobial susceptibility, Molecular characterization

## Abstract

**Background:**

Emergence and spread of β-lactamase resistant *Klebsiella pneumoniae* have posed *a* serious threat, especially in paediatric patients globally. The present study focuses on explore drug resistance profile and molecular characterization of carbapenemase and extended-spectrum β-lactamase producing *K. pneumoniae* isolated from paediatric patients in Shenzhen, China.

**Methods:**

Present study, a total of 31 isolates of multi-drug resistant *K. pneumoniae* were collected from Shenzhen Children’s Hospital, China during Jan 2014 to December 2015. ESBLs production was confirmed by using the combination disc diffusion method followed by antimicrobial susceptibility. In addition, β-lactamase encoding genes were determined by PCR assay and sequencing. The genotypic diversity and phylogenetic relationship were determined by multi-locus sequence typing (MLST) method and pulsed-field gel electrophoresis (PFGE).

**Results:**

We examined 31, unique *K. pneumoniae* isolates collected from 2014 and 2015 in Shenzhen Children’s Hospital, China. All the 31 isolates 100% were resistant to ceftazidime, ertapenem, ampicillin, cefazolin and ampicillin-sulbactam followed by ceftriaxone 94% (n = 29), aztreonam 89% (n = 26), cefepime 84% (n = 26), nitrofurantoin 75% (n = 24), piperacillin 52% (n = 16), and levofloxacin 49% (n = 15). Of the 31 β-lactamase gene coding isolates, *bla*_CTX-M_ was mainly detected in about 100% (n = 31), followed by *bla*_KPC_ 71% (n = 22), *bla*_SHV_ 61% (n = 19), *bla*_NDM_ 25% (n = 8), *bla*_CYM_ 13% (n = 4), *bla*_OXA-48_ 9% (n = 3), *bla*_GES_ 9% (n = 3) and *bla*_TEM_ 6% (n = 2). Seventeen distinct sequences type were observed with ST20 being mostly identified 16% (n = 5). Pulsed-field gel electrophoresis (PFGE) typing showed that identical profile for the isolates recovered from the Department of Intensive Care Unit and Department of Neurology of our hospital. Plasmid replicon typing result indicates the presence of IncFIS type as highest in all isolates as 61% (n = 19), followed by IncFIB 23% (n = 7), IncFIA and IncFIC 16% (n = 5) each.

**Conclusion:**

Our study reports the occurrence and spread of extended β-lactamase *K. pneumoniae* ST20 and ST2407 for the first time, in Shenzhen, particularly in paediatric patients. To prevent and control the infection by limiting the spread of infection-causing organisms it is very crucial to detect the presence of resistant genes at an early stage.

## Introduction

*Klebsiella pneumoniae* (*K. pneumoniae*) has most common pathogen intricate in healthcare-associated infections particularly in children that cause a wide variety of infections consisting pneumonia, urinary tract infections, bloodstream infections, intra-abdominal infection and bacteraemia and such conditions treat by antibiotics [1–3]. Indiscriminate use of antibiotics has led to the global spread of extended-spectrum β-lactamases (ESBLs) and *K. pneumoniae* carbapenemase (KPC) in *K. pneumoniae* being reported globally [4]. ESBLs have the ability to hydrolyse broad spectrum β-lactams antibiotics including cephalosporin. Since 1983, ESBLs-producing *K. pneumoniae* infections have been proved to be difficult to treat. ESBLs encoding genes such as *bla*_SHV_, *bla*_CTX-M_, *bla*_TEM_, *bla*_OXA_, *bla*_PER_, and *bla*_VEB_ were generally reported from such infections. Eventually, several genes encoding for carbapenemase have also been reported in *K. pneumoniae* which includes class-A β-lactamase *K. pneumoniae* carbapenemase (KPC), class-B β-lactamases, IMP, VIM, New Delhi Metallo-β lactamase (NDM) and class-D β-lactamase-oxacillinase type 48 (OXA-48) [5]. Co-production of KPC and NDM in *K. pneumoniae* was reported to have notably increased in Canada, USA, and China has reported a notable increase in the co-occurrence of KPC and NDM in *K. pneumoniae* while Africa reported having a major spread of OXA-48 like producing strains [6]. Among paediatric cases from China, only NDM-producing *K. pneumoniae* is primarily reported in maximum cases despite the presence of wide-spread KPC-producing strains [7]. According to the CHINET antimicrobial surveillance program conducted for 2005–2014, reports of carbapenem-resistant *K. pneumoniae* have increased from 5.3 to 15.9% in paediatric patients in China [8]. The co-harbouring of two or more clusters of resistance genes in *K. pneumoniae* have added to poses a challenge for prescribing efficient antibiotic medication. Unfortunately, the lack of effective treatment therapy and extensive application of invasive treatment methods have resulted in the rise of the infections caused by multi-drug resistant *K. pneumoniae*, ultimately causing mortality in some cases [9, 10]. Co-existence of ESBLs and KPC in *K. pneumoniae* has world-wide dissemination causing life-threatening clinical outcomes in pediatric patients however, very no more data is available on the susceptibility and molecular characteristics of this pathogen in China. Identification of drug resistance genes and pattern of resistance aids in the targeted drug use which acts as a barrier for further spread of the infection. Hence, we propose to study phenotype and genotype of multi-drug resistant *K. pneumoniae* isolates obtained from paediatric clinical cases which are first of its kind study in the Shenzhen, China.

## Materials and methods

### Bacterial isolation and identification

Thirty-one non-duplicate (one isolate from one patient) clinical isolates of *K. pneumoniae* were collected during January 2014 to December 2015 from Shenzhen Children’s Hospital. The hospital comprises of 1220 beds, offering state of the art health facilities to the surrounding population of the southern part of China. Among the 31 ESBLs producing *K. pneumoniae* isolates 17 (55%) were from male and 14 (45%) were from female, patients, with age ranging from 1 month to 12 years. The clinical isolation site for specimens were as follows, sputum n = 11, blood n = 6, urine n = 5, catheter-associated and stool n = 3 each, cerebral-spinal fluid n = 2 and throat swab n = 1 (Fig. 2). All isolates were primarily identified by VITEK@2-(Biomerieux, Ref. No. 27530/275660) automated system and confirmed by using 16s RNA gene sequencing.

### Phenotypic detection of ESBLs production

The combination disc test was done for phenotypic detection of ESBLs production. The test was performed by using a disc of both cefotaxime and ceftazidime, separately and in combination with clavulanic acid. Control strain, which was selected from the characterized strain collection of our laboratory while ATCC25922 used as a negative control strain. The ESBLs production result was analysed as per the Clinical and Laboratory Standards Institute (CLSI) guideline (CSLI, 2010).

### Antimicrobial susceptibility test

Antimicrobial susceptibility was performed by using VITEK@2 compact system (Biomerieux, Ref. No. 27530/275660) for 18 antimicrobial agents namely, ampicillin/sulbactam, piperacillin, ertapenem, amikacin, levofloxacin, nitrofurantoin, ampicillin, cefazolin, ceftazidime, ceftriaxone, cefepime, imipenem, cefotetan, tobramycin, gentamicin, and ciprofloxacin. The results were construed according to the Clinical and Laboratory Standards Institute (CLSI) guideline (CSLI, 2010).

### Detection of β-lactamase genes

The standard PCR was performed to detect the presence of ESBLs producing genes: *bla*_TEM_, *bla*_SHV_, *bla*_CTX-M (variant)_, *bla*_GES_, *bla*_CYM_ and *bla*_VEB_ using specific primers previously described [11]. Carbapenemase genes including *bla*_KPC_, *bla*_NDM_, and *bla*_OXA_ were detected by PCR as previously described [12]. The purified PCR products were sequenced commercially (Sangon Biotech-Shanghai, China). DNA Sequences were analysed by (https://blast.ncbi.nlm.nih.gov/Blast.cgi) and (https://bigsdb.pasteur.fr/klebsiella/klebsiella.html).

### PFGE and multi-locus sequence typing (MLST)

We performed PFGE to check whether there is the presence of any clonal transmission within the hospital and MLST was applied to assess the genetic relatedness of the identified isolates. Post extraction, DNA was digested with 45U Xbal (Takara Biotech) for 2 h at 37 °C. We used CHEFDRIII apparatus (Bio-Rad Laboratories, Hercules, CA, USA) to perform PFGE for *K. pneumoniae* isolates as per earlier described [13]. Amplified fragments of seven housekeeping genes (gapA, infB, mdh, pgi, phoE, rpoB, and tonB) were sequenced to MLST analysis and MLST website MLST website-http://www.pasteur.fr/recherche/genopole/PF8/mlst/Kpneumoniae was referred to assign the STs.

### Plasmid transferability

Streptomycin-resistant *E. coli* C_600_ used as the recipient strain in conjugation experiments to analyse the horizontal gene transfer of *bla*_CTX-M_ and *bla*_KPC_ for ESBLs and carbapenemase producing respectively in *E*. *coli* isolates. We used a liquid mating assay as described earlier [14]. Transconjugants were selected Luria–Bertani agar containing streptomycin 2000 (µg/ml) and cefotaxime (32 µg/ml). Transconjugants were further tested for ESBLs and KPC enzyme production after performing a phenotypic test.

### PCR-based replicon typing

PCR-based replicon typing was performed for both plasmids from parental and transconjugant isolates. The Inc (incompatibility) groups were determined by using specific primer introduced by Carattoli et al. [15].

## Results

### Antimicrobial resistance profile of *K. pneumoniae*

**Fig. 1:**
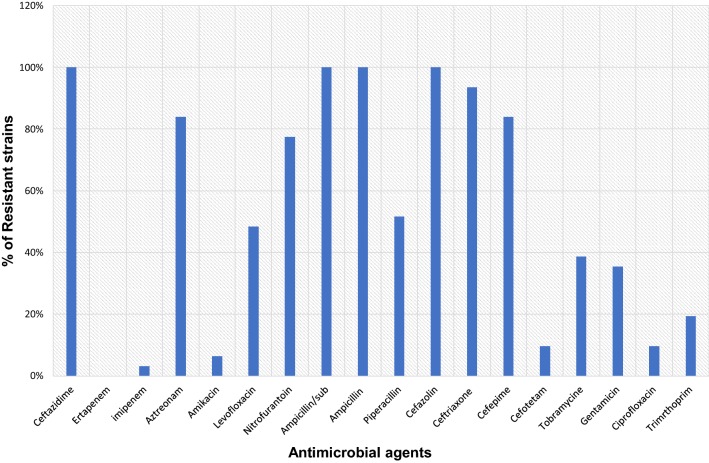
Antimicrobial susceptibility of 31 extended spectrum β-lactamase producing *Klebsiella pneumoniae* isolates recovered from paediatric patients to commonly used antibiotics

All the 31 isolates were confirmed as *K. pneumoniae* by VITEK@2-(Biomerieux, Ref. No. 27530/275660) automated system and by using 16s RNA gene sequencing. Primarily all the isolates were confirmed for ESBLs production by combination disc test followed by susceptibility. Antimicrobial susceptibility tests reflected that all of the 31 ESBLs producing *K. Pneumoniae* isolates 100% were resistant to ceftazidime, ertapenem, ampicillin, cefazolin and ampicillin-sulbactam followed by ceftriaxone 94% (n = 29), aztreonam 89% (n = 26), cefepime 84% (n = 26), nitrofurantoin 75% (n = 24), piperacillin 52% (n = 16), levofloxacin 49% (n = 15), tobramycin 39% (n = 12), gentamycin 35% (n = 11), trimethoprim 19% (n = 6), cefotetan and ciprofloxacin 10% (n = 3) each and amikacin 6% (n = 2). However, all of the isolates were susceptible to imipenem (Fig. 1). All the 30 ESBLs encoding gene *K. pneumoniae* isolates exhibited a multi-drug resistant phenotype (resistant to at least one agent in three or more antimicrobial group), hence proves the term ‘superbugs’.

### Molecular analysis of drug resistance genes

**Fig. 2:**
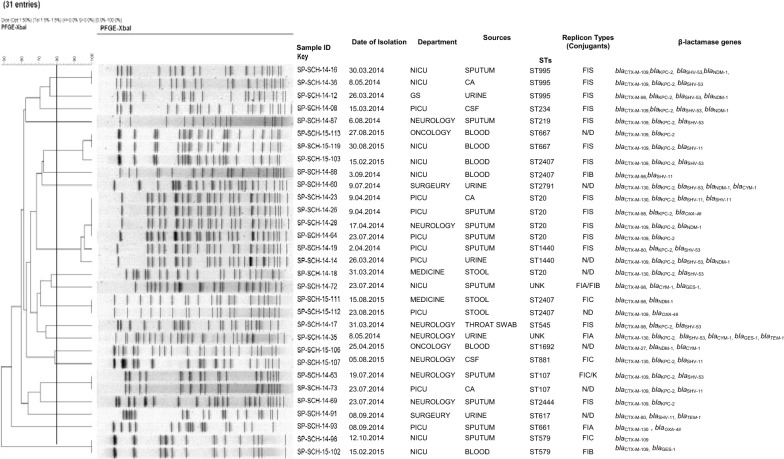
Dendrogram of the 31-PFGE-Xbal identified extended spectrum β-lactamase producing *Klebsiella pneumoniae* isolates recovered from paediatric patients showing their clustering by date of isolation, department of isolation, sources of specimen, sequences types, producing beta-lactamases and clonal relatedness

All of the 31 ESBLs-genes encoding *K. pneumoniae* isolates were carrying *bla*_CTX-M_ genes, with the most common being *bla*_CTX-M-109_ (52%, n = 16), followed by *bla*_CTX-M-130_ (19%, n = 6) and *bla*_CTX-M-98_ (19%, n = 6), *bla*_CTX-M-80_ (7%, n = 2) and *bla*_CTX-M-27, 109_ (3%, n = 1). Additionally, co-existence of other β-lactamase genes were detected, *bla*_KPC-2_ (71%, n = 22), *bla*_SHV-53_ (42%, n = 13), *bla*_SHV-11_ (19%, n = 6), *bla*_NDM-1_ (25%, n = 8), *bla*_CYM-1_ (13%, n = 4), *bla*_OXA-48_ (9%, n = 3), *bla*_GES-1_ (9%, n = 3) and *bla*_TEM_ (6%, n = 2) (Fig. 2). The *bla*_VAB_ gene was not find in this study. There was no noteworthy difference in the occurrence of *bla*_CTX-M_ genes among the ESBLs-producing *K. pneumoniae* isolated from the different isolation sites or even samples.

### Multi-locus sequences typing and PFGE

The extensive diversity of MLST was recorded from ESBLs producing *K. pneumoniae* isolates, with a total of 17 different STs of which, ST20 (16%) was highly prevalent in Shenzhen, China. All the ST20 isolates were recovered from the Department of Neurology and Department of Intensive Care Unit, the results indicated that *K. pneumoniae* ST20 is dominant in these two wards and a key transporter for the *bla*_CTX-M-109_ gene. Our particular concern is that the *bla*_CTX-M-109_ gene was reported in nine different STs in Shenzhen Children’s Hospital. This observation suggests that ESBLs-producing *K. pneumoniae* isolates carrying *bla*_CTM-M-109_ gene might have spread in the Shenzhen region and may be widespread in Southern China. The 31 ESBLs-producing *K. pneumoniae* isolates were allocated to 20 distinct PFGE clusters sharing ≥ 80% band similarity. The PFGE results showed that the clonal transmission was often observed in the same department and within different departments in the hospital (Fig. 2).

### Plasmid profiling

The successful transconjugants were selected from Luria–Bertani agar containing streptomycin 2000 (µg/ml) and cefotaxime (32 µg/ml). PCR based replicon type assay results showed that Inc (Incompatibility) plasmids groups IncFIS (n = 14), IncFIC (n = 4), IncFIB and IncFIA (n = 3) each, were carrying the *bla*_CTX-M_ group gene (Fig. 2). Conjugants were not obtained from seven parental strains.

## Discussion

ESBLs producing *K. pneumoniae* is a serious threat to pediatric patients, particularly in new-borns, owing to restricted therapeutic options [16]. This pathogen is now considered a reservoir for virulence and resistance genes due to the acquisition of β-lactamase and recently reported colistin resistance *mcr*-*1* gene and so make this a serious threat to human and animal [17, 18]. Our study revealed that CTX-M109 was the most prevalent β-lactamase in Shenzhen, China. So far, no data available on ESBLs producing *K. pneumoniae* in paediatric clinical cases particularly those caused by CTX-M109 producing *K. pneumoniae.* In our study, we first determined the phenotype and genotype of multi-drug resistant *K. pneumoniae* isolates obtained from the paediatric clinical cases in Shenzhen, China. The present study showed that *K. pneumoniae* were highly resistant to commonly used antibiotics, except for imipenem, amikacin and cefotetan. We did not find any significant antimicrobial resistance profile among the ESBLs producing *K. pneumoniae* which is different from studies in Shanghai [19]. We found *bla*_CTX-M-109_ as the predominant genotype of ESBLs-producing *K. pneumoniae* in Shenzhen followed by *bla*_CTX-M-9_, *bla*_CTX-M-130_, *bla*_CTX-M-80_, and *bla*_CTX-M-27_ revealing the diversity of CTX-M genotype of ESBLs producing *E. coli* in Shenzhen, China. Similar results were reported from across China [20]. We first report the high presence of co-existing carbapenem-resistant genes *bla*_KPC-2_ (71%), *bla*_NDM-1_ (25%) with ESBLs encoding gene in Shenzhen, China as well co-existence of carbapenem resistance genes in ESBLs-producing *K. pneumoniae* has flagged concern about the spread of such superbugs in the Shenzhen area. Several reports have shown the co-existence of carbapenem resistance genes and ESBLs encoding genes in *K. pneumoniae* on a global scale [21]. Our MLST results demonstrated that ST20 and ST2407 were highly prominent in Shenzhen area which encodes ESBLs genes. Several countries such as New Zealand [22], Greece [23], Canada [5], Korea [24] have reported infection caused by *K. pneumoniae* ST20 with ESBLs production. Jin et al. first reported an outbreak of ESBLs producing *K. pneumoniae* ST20 during August 2012 to September 2013, among neonates in Shandong province, China [25]. We find the clonal transmission within the hospital, which is comparable with previous studies by Dongxing Tian et al., in 2018 at Shanghai, China [19]. The transmission may occur due to direct contacts with the patients or hospital staff (hands, saliva, other body fluids etc.) or environmental sources such as water, food, other body fluids. We strictly follow the standard operating procedure for patient handling in our hospital to restrict such superbugs transmission. The limitation of our study is that we did not study isolates from other hospitals to observe whether there is clonal transmission between the hospitals. Plasmid replicon typing and conjugation experiment results exposed that IncFIS, IncFIB and IncFIA replicons were existing in the transconjugants and *bla*_CTX-M_ genes co-transfer with carbapenemase coding genes such as *bla*_KPC-2_, and *bla*_NDM-1_. No apparent relationship between replicon and sequence type was observed among the current isolates. We did not the dissemination of any particular clones in Shenzhen, China. Our finding stresses the significance of continuous monitoring to detect multi-drug resistant isolates so as to promote therapeutic strategies for treating infections in paediatric patients. We propose a new study to assess the presence of other resistance-related determinants, such as the outer-membrane permeability and also to augment sample size for the molecular study.

## Conclusion

Earlier studies in China have reported the occurrence of ESBL-producing *K pneumoniae* however, information regarding the susceptibility pattern and description of molecular structure in paediatric patients is very limited. Best of our knowledge, For the first time, we report the emergence of ESBLs from a major Children’s Hospital in Shenzhen, China. Our results highlight the occurrence of ESBLs in *K. pneumonia*e belonging to the CTX-M-109 type.

## Data Availability

Not available.

## Abbreviations

MLST: multi-locus sequence typing (MLST) method and (PFGE)
PFGE: pulsed-field gel electrophoresis
ESBLs: extended-spectrum beta-lactamases
Inc: incompatibility
STs: sequences types
